# Evaluation of Medication Use Pattern Among Patients Presenting to the Emergency Department of Hiwot Fana Specialized University Hospital, Using WHO Prescribing Indicators

**DOI:** 10.3389/fphar.2020.00509

**Published:** 2020-04-28

**Authors:** Kirubel Minsamo Mishore, Yabsira Girma, Assefa Tola, Abraham Nigussie Mekuria, Yohanes Ayele

**Affiliations:** ^1^Department of Clinical Pharmacy, School of Pharmacy, College of Health and Medical Sciences, Haramaya University, Harar, Ethiopia; ^2^Department of Pharmacy, Janmeda Health Center, Addis Ababa, Ethiopia; ^3^Department of Epidemiology and Biostatistics, School of Public Health, College of Health and Medical Sciences, Haramaya University, Harar, Ethiopia; ^4^Department of Pharmacology, School of Pharmacy, College of Health and Medical Sciences, Haramaya University, Harar, Ethiopia

**Keywords:** drug utilization, emergency department, WHO core indicator, Ethiopia, evaluation

## Abstract

**Background:**

Ensuring rational drug use requires ongoing evaluation of drug prescribing, dispensing, and use by patients. Health care providers working in an emergency department face unique challenges, including making urgent decisions, patient overload, and limited resources, which contribute to inappropriate drug use. Rational medication use should be an important aspect of emergency care to improve patient outcomes. Thus, this study was conducted to assess medication utilization patterns using World Health Organization (WHO) prescribing indicators in the emergency department.

**Methods:**

A cross-sectional study design was implemented among patients presenting at the emergency department of Hiwot Fana Specialized University Hospital (HFSUH) from January to March 2018. The data were collected from the medical charts of a total of 342 patients using a pre-prepared structured format according to WHO recommendations. The data were analyzed using SPSS version 21 software and presented in tables and figures.

**Results:**

The most commonly reported clinical diagnosis was found to be soft tissue laceration or abrasion, in 75 patients (21.9%), followed by dyspepsia, in 50 (14.6%), and severe pneumonia, in 44 (12.9%). A total of 810 drugs were prescribed for the 342 patients. The main category of drugs prescribed were analgesics, constituting 125 (29.2%), followed by antibiotics, 120 (28.0%). Regarding WHO prescribing indicators, the average number of drugs prescribed per encounter was 2.36, the number of encounters at which antibiotics were prescribed was 127 (37.13%), and injections were prescribed at 300 (87.7%) encounters. All of the drugs prescribed were from the National Essential Medicine List (NEML) of Ethiopia, and 780 (98.1%) of the drugs were prescribed by international nonproprietary name.

**Conclusion:**

Overall, there were inflated use of antibiotics and injection drugs, whereas prescribing by international nonproprietary name and prescribing from NEML were according to the recommendations. Hence, the hospital should work to ensure the judicious use of antibiotics and injection drugs.

## Introduction

Medicines are an essential component of health care delivery. When used rationally, they produce the desired effect of improving patients’ ailments. Their irrational use, on the other hand, leads to prolongation of the illness, development of adverse effects, and unnecessary expense. In Ethiopia, similar to other low- and middle-income countries, 70 to 75% of the total budget of health care is allocated to the pharmaceutical sector (FMHACA, 2012a, Cameron et al., 2009). The health system in Ethiopia is structured into three tiers: referral, general, and primary hospitals. Referral hospitals provide specialized and sophisticated forms of care, whereas general hospitals provide inclusive services, and primary health care units operate at the district and kebele (lowest administrative) levels, providing first-level services to the local communities (FDRE MOH, 2010).

Irrational drug use refers to the use of drugs when they are not needed. It also means prescribing drugs without adequate scrutiny regarding their efficacy, safety, affordability, and suitability to the patient. Many countries are doing their best to curb the problem of irrational drug use by developing national programs that promote appropriate prescribing behavior (Ofori-Asenso and Agyeman, 2016). In Ethiopia, several efforts have been made to promote the rational use of drugs. The most noticeable action was the preparation of guidelines and manuals for the health care sectors, for example, the National Essential Medicine List for Ethiopia (FMHACA, 2014a), Standard Treatment Guidelines for hospitals (FMHACA, 2014b), Manual for Medicine Good Prescribing Practice (FMHACA, 2012a), and Manual for Good Medicine Dispensing Practice (FMHACA, 2012b). Furthermore, there were also notable efforts to establish and strengthen Drug and Therapeutic Committees and Drug Information Centers in various hospitals. However, the contribution of the latter component is still minimal as there is no full implementation of such services in hospitals.

Although the commendable efforts of different stakeholders in the country have produced encouraging results, there is evidence indicating the irrational use of drugs in Ethiopia. A baseline survey conducted in different parts of Ethiopia identified irrational prescribing in the form of a high average number of drugs per encounter and high percentages of injection drug and antibiotic prescriptions (Dilbato et al., 1998; Sisay et al., 2017).

Ensuring rational drug use requires that there is ongoing evaluation of drug prescribing, dispensing, and use by patients. However, evaluation of drug use is not an easy task, as the use of drugs is influenced by many factors that are often difficult to measure and quantify (Bachhav and Kshirsagar, 2015). Nevertheless, a number of tools have been developed, standardized, and evaluated by the WHO for the evaluation of drug utilization. Particularly, the tools called “WHO drug use indicators” are the most popular in this regard. These indicators are grouped into three categories, namely: prescribing indicators, patient care indicators, and facility indicators. Prescribing indicators include the average number of drugs per encounter, the percentage of encounters in which antibiotics are prescribed, the percentage of encounters in which an injection is prescribed, the percentage of drugs prescribed by generic name, and the percentage of drugs prescribed from an essential drug list or formulary (WHO, 1993).

Emergency care is a process of delivering care for a patient who is in urgent need and is often in critical condition (Fatovich, 2002). In this setting, health care providers are expected to make quick decisions, often with high precision, as this can make the difference between life and death for the patient. Other factors such as heavy patient load and limited resources, particularly in developing countries, are another challenge health care providers face in this department (Patidar and Pichholiya, 2016; Yarmohammadian et al., 2017). As a result, physicians are struggling to select, initiate, and individualize appropriate drug therapy for patients admitted in the emergency medicine ward, not to mention the problems associated with administration (Peth, 2003; Dalen, 2010; Cheekavolu et al., 2011; Dabaghzadeh et al., 2013; Vazin et al., 2014; Di Simone et al., 2018). For example, a study reported over 50% medication errors in this department (Dabaghzadeh et al., 2013). Moreover, the prescribing behavior of physicians can be influenced by different factors such as peer-norms, lack of drug information, workload, and availability of financial incentives (Dilbato et al., 1998; Reynolds and Mckee, 2011; Li et al., 2012; Zeng et al., 2015; Soleymani et al., 2019).

Rational medication use should be an important aspect of emergency care to improve patient outcome. To this end, a periodic evaluation of drug use should be conducted to ensure that the hospitals are utilizing drugs according to recommended standards. In addition, though there have been various studies conducted across the country in different wards, the emergency department is often overlooked, and, consequently, less is known about drug use in this ward. The consequences of facility-related factors in availing vital drugs for emergency cases might be different from the case of the use of other drugs for treatment in outpatient departments. Hence, we believe that conducting this kind of study will serve to offer insight into current practice and improve upon it in the future. Therefore, the aim of this study was to evaluate the medication use pattern using WHO prescribing indicators among patients presenting to the emergency department of HFSUH.

## Materials and Methods

### Study Setting, Design, and Participants

This study was conducted in Harar city, which is located about 525 km away from the capital, Addis Ababa, in the eastern direction. HFSUH serves as a referral hospital for the entire eastern part of Ethiopia, including Eastern Oromia, Dire Dawa City Administration, and the Somali and Harar Regional States. The hospital has various departments, including emergency, internal medicine, surgery, pediatric, gynecology, and obstetrics departments. The present study was conducted at the emergency department from January to March 2018. A descriptive cross-sectional study was employed to assess the drug utilization pattern using WHO prescribing indicators.

### Sample Size and Sampling Technique

The sample size was determined by using a single proportion formula for cross-sectional survey and taking the proportion of the prevalence of the rate of drug utilization as 50%, with a confidence level of 95% and a degree of precision of 5%. By considering the total number of admissions during the study period, a finite population correction formula was used, and the final sample size was 342. The medical records of 342 patients were randomly selected from the emergency department of HFSUH.

### Data Collection Tools and Procedure

Data were collected by two trained nurses who have BSc degrees. The pre-tested structured format was used to collect the data by reviewing patients’ medical records. The format was developed based on the WHO prescribing indicators and by reviewing related studies (WHO, 1993; Demeke et al., 2015; Sisay et al., 2017). The format developed was pretested in the emergency department of Jugal hospital (another government hospital in Harar city), and subsequent correction was done. The format contains socio-demographic variables, presenting complaint, diagnosis, and complete prescription. The data collectors were trained on the data-collection technique. The collected data were reviewed and checked for completeness before data analysis.

### Data Processing

The collected data were coded, entered, cleaned, and analyzed by using SPSS version 21. Descriptive analyses such as percentage and frequency distributions were performed. WHO prescribing indicators were calculated, including the average number of drugs per encounter, percentage of drugs prescribed by international nonproprietary name, percentage of drugs prescribed from the essential drug list, and percentage of encounters at which antibiotics were prescribed. Finally, the result was interpreted and presented in tables and graphs.

### Operational Definitions

#### Average Number of Drugs Per Encounter

The average number of drugs prescribed per encounter is calculated to measure the degree of poly-pharmacy. It was calculated as

$$\text{Average number of drugs per encounter}=\frac{\text{total number of different drug products prescribed}}{\text{number of encounters surveyed}}$$

Combinations of drugs prescribed for one health problem will be counted as one.

#### Percentage of Drugs Prescribed by International Nonproprietary Name

Percentages of drugs prescribed by international nonproprietary name are calculated to measure the tendency to prescribe by international nonproprietary name. It was calculated as

$$\text{Percentage of drugs prescribed by international nonproprietary name}=\frac{\text{number of drugs prescribed by international nonproprietary name}}{\text{total number of drugs prescribed}}x100$$

#### Percentage of Encounters With Injection Prescribed

The percentage of encounters at which an injection was prescribed is calculated to measuring the overall use level of commonly overused and costly forms of drug therapy. It was calculated as

$$\text{Percentage of encounters with injection prescribed}=\frac{\text{number of patients encounters in which an injection was prescribed}}{\text{total number of encounters surveyed}}X100$$

#### Percentage of Encounters With Antibiotics Prescribed

The percentage of encounters in which an antibiotic was prescribed is calculated to measure the overall use of commonly overused and costly forms of drug therapy. It was calculated as

$$\text{Percentage of encounters with antibiotics prescribed}=\frac{\text{number of patients encounters in which an antibiotics was prescribed}}{\text{total number of encounters surveyed}}X100$$

#### Percentage of Drugs Prescribed From Essential List or Formulary

The percentage of drugs prescribed from NEML is calculated to measure the degree to which practices conform to national drug policy as indicated in the national drug list of Ethiopia. The percentage was calculated as

$$\text{Percentage of drugs prescribed from essential list of formulary}=\frac{\text{number of products prescibed that are in essential drug list}}{\text{total number of drugs prescribed}}X100$$

### Ethical Considerations

Initially, ethical clearance was obtained from the School of Pharmacy, College of Health and Medical Sciences, Haramaya University. Patients’ data were accessed upon the approval of the medical director of HFSUH. Confidentiality was ensured during the data collection, and the information gathered for this study was not disclosed to others.

## Results

### Socio-demographic Characteristics of Patients

The medical records of a total of 342 patients were reviewed. Among these, 194 (58.7%) were males, and the remaining were females. The mean (± SD) age of patients admitted to the emergency department was 37.9 ± 12.5 years. A significant proportion of patients (60.2%, 206 patients) presented to the emergency department were from rural areas (**Table 1**).

**Table 1:** Socio-demographic characteristics of patients admitted to the emergency department of HFSUH, Harar, Ethiopia, 2018 (n = 342).

Variable	Category	N (%)
Sex	Male	194 (58.7)
	Female	148 (43.3)
Age (in years)	≤20	10 (2.9)
	21-30	124 (36.3)
	31-40	68 (17.8)
	41-50	81 (23.3)
	≥50	59 (17.2)
Residence	Urban	13 (39.8)
	Rural	206 (60.2)

### Clinical Characteristics of Patients

**Figure 1:**
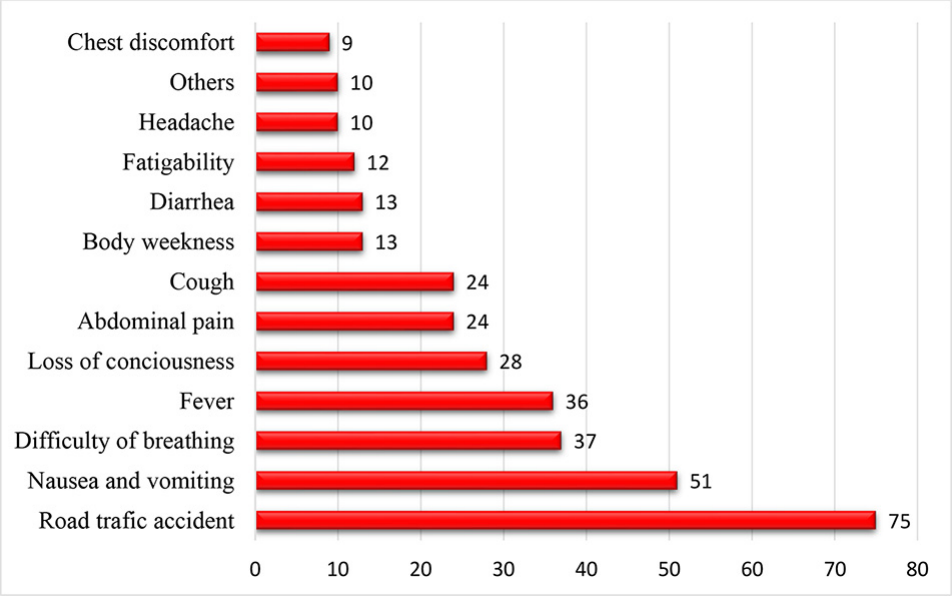
Chief cause of admittance to the emergency department of HFSUH Harar, Ethiopia, 2018 (n=342).

Out of 342 patients, the most common chief cause of admittance to the emergency department was trauma and accident (75 patients, 21.9%), followed by nausea and vomiting (51 patients, 14.9%) (**Figure 1**).

Regarding the specific clinical diagnosis stated in the medical records, the most common associated clinical diagnosis was found to be soft tissue laceration or abrasion, in 75 (21.9%), followed by dyspepsia, in 50 (14.6%), and severe pneumonia, in 44 (12.9%) (**Table 2**). We also assessed the duration of stay in the emergency department and found that the average duration was 19.6 ± 14 hours. The majority of the patients improved and were discharged to their homes (73.1%), and 26.9% of patients were transferred to other wards (**Table 2**).

**Table 2:** Specific clinical diagnosis of patients admitted to the emergency department of HFSUH, Harar, Ethiopia, 2018(n = 342).

Clinical diagnosis	n(%)
Soft tissue laceration or abrasion	75(21.9)
Dyspepsia	50(14.6)
Severe pneumonia	44(12.9)
Pyelonephritis	35(10.2)
Acute gastroenteritis	20(5.8)
Stroke	20(5.8)
Poisoning	16(4.7)
Asthma	15(4.4)
Congestive heart failure with pulmonary edema	15(4.4)
Severe anemia	12(3.5)
Hypertensive crises	10(2.9)
Acute Coronary Syndrome	10(2.9)
Burn	10(2.9)
Others*	10(2.9)

*Ketoacidosis, hyperemesis gravidarum, Acute pancreatitis, COPD (chronic obstructive pulmonary disease

### Drugs Prescribed in the Emergency Department

The main drugs prescribed were analgesics, at 125 prescriptions (29.2%), Diclofenac (72, 21.1%) and Tramadol (53, 15.4%) being the most common. This was followed by antibiotics (120, 28.0%) such as cloxacillin (27, 7.9%), ciprofloxacin (35, 10.2%), ceftriaxone (28, 8.2%), Azithromycin (16, 4.7%), and metronidazole (14, 4%). Anti-ulcer medicines (76, 17.8%), such as cimetidine (42, 12.3%) and omeprazole (18, 5.3%), and anti-hypertensive medication (30, 8.7%), such as hydralazine (10, 2.9%) and nifedipine (20, 5.8%), were also commonly prescribed agents (**Table 3**).

**Table 3:** Commonly prescribed classes of drugs for patients admitted to the emergency department of HFSUH, Harar, Ethiopia, 2018 (n = 433).

Commonly used drugs	n (%)
Analgesics	125 (29.2)
Antibiotics	120 (28.0)
Antiulcer	76 (17.8)
Antihypertensive	30 (7.0)
Antiplatelet	22 (5.2)
Diuretics	15 (3.5)
Inhaled bronchodilator	15 (3.5)
Drugs for poisoning	10 (2.3)
Others*	15 (3.5)

*Anti-emetics, corticosteroids, vitamins and minerals, anti-constipation drugs, oral rehydration solution.

### WHO Drug Prescribing Indicators in the Emergency Department of HFSUH

The average number of drugs per encounter was 2.36. The total number of drugs prescribed by international nonproprietary name was 795 (98.1%). Antibiotics were prescribed in 127 (37.13%) encounters, and injections were prescribed in 300 (87.7%) encounters. All drugs prescribed were from the EDL of Ethiopia (**Table 4**).

**Table 4:** Drug prescribing indicators for the emergency department of HFSUH, Harar, Ethiopia 2018 (n = 342).

Prescribing indicator	Average/percentage
Average number of drugs per encounter	2.36
Percentage of encounters with antibiotics prescribed	37.13%
Percentage of encounters with injection prescribed	87.7%
Percentage of drugs prescribed by international nonproprietary name	98.11%
Percentage of drugs from NEML	100%

## Discussion

In this study, the drug utilization pattern of the emergency department a tertiary care hospital, HFSUH, was evaluated by focusing on WHO prescribing indicators. Accordingly, the average number of drugs prescribed per encounter, percentage of drugs prescribed by international nonproprietary name, percentage of drugs prescribed from the NEML, percentage of encounters at which antibiotics were prescribed, and percentage of encounters at which injections were prescribed were assessed.

In the present study, the average number of drugs prescribed per encounter was 2.36, which was slightly greater than the value indicated in the WHO guideline (1.6–1.8) (Isah AO; WHO, 1993). Although there is deviation from the recommended value, the use of more than two drugs at a time could be justifiable in this setting, as there is a need for empirical therapy until definitive diagnosis is made and the patients may require more than two drugs for management of acute life-threatening conditions. However, it is always preferable to keep the number of drugs per prescription as low as possible to minimize adverse effects and drug interactions and to reduce the cost of therapy. This finding is encouraging compared to studies conducted elsewhere such as Nigeria (Erah et al., 2003), Ghana (Baba Sulemana Mohammed, 2019), Oman (Al Balushi et al., 2013), and India (Cheekavolu et al., 2011; Kaur et al., 2014). The result is also significantly lower than a finding from a systematic review conducted by Ofori-Asenso, R. et al., who reported an average number of medicines prescribed per encounter of 3.1 (Ofori-Asenso et al., 2016). On the other hand, the figure is slightly higher than has been reported from Southern Ethiopia (Desalegn, 2013; Gidebo et al., 2016). This deviation might be due to the difference in setting, where the studies conducted as the latter study evaluated the use of drugs across all wards of the hospital.

Regarding antibiotics, in the current study, the percentage of encounters where antibiotics were prescribed was 37.13%. Appropriate use of antibiotics is necessary to prevent the emergence of drug-resistant microorganisms. However, this result is moderate compared to other studies conducted in Ethiopia (Sisay et al., 2017a, Desalegn, 2013) and other African countries (Ofori-Asenso et al., 2016; Baba Sulemana Mohammed, 2019), which reported a high prevalence of antibiotics use. This lower value in the emergency department can be explained by the nature of the patients treated in this ward. In this setting, the most common diagnosis was soft tissue laceration or abrasion (75 patients, 21.9%) followed by dyspepsia (50, 14.6%), which are often treated with analgesic and antiulcer drugs, respectively. Nevertheless, this finding shows considerable deviation from the WHO standard, which recommends less than 20–26.8% of antibiotic use in countries where infectious diseases are prevalent (WHO, 1993). It is also significantly higher than found by studies conducted in other countries (Shakirat Iyabo Bello et al., 2016; Denny et al., 2018). This high prevalence of antibiotics use could be due to a lack of regulatory systems at the hospital level, such as restrictions on antibiotics access (Chokshi et al., 2019).

In our study, the percentage of encounters where an injection was prescribed was 87.7%. This result is much higher than in studies conducted elsewhere, for example in Ghana, 14% (Baba Sulemana Mohammed, 2019), in a systematic review on 11 African countries, 25.0 % (Ofori-Asenso et al., 2016), in Saudi Arabia, 23% (Ahmed Alkahtani, 2018), in Yemen, 46.0% (Bashrahil, 2010), and in Oman, 38% (Al Balushi et al., 2013). What is more, the percentage is markedly higher than the WHO recommendation (13.4%-24.1%) (WHO, 1993). Different factors contribute to an increased tendency to use injection drugs. The physicians’ high opinion of the effectiveness of parenteral therapy, demand for injections from patients, and incentives for physicians to prescribe injections are often reported as driving factors (Hwang et al., 2007; Chowdhury et al., 2011; Reynolds and Mckee, 2011; Li et al., 2012; Zeng et al., 2015; Yousefi et al., 2017). However, the high injection use in this study setting can be explained by the physical need for rapid effect. Furthermore, some of the patients are critically ill and require parenteral therapy because they cannot take oral doses. Although it might be rational to choose injection in the emergency department to a certain extent in order to ensure rapid and predictable effect, unjustifiable use of injection should be discouraged.

Prescribers are encouraged to prescribe by international nonproprietary name since it enables the selection between more alternatives and has a major impact in terms of cost minimization. In this study, the percentage of drugs prescribed by international nonproprietary name was 98.1%, which is comparable with the WHO recommendation (100%). Furthermore, the percentage of drugs prescribed from the NEML was satisfactory. According to the result of this study, all of the drugs were prescribed from the NEML of Ethiopia, which is in line with the WHO recommended level, 100%. This report is also in agreement with a study conducted in another part of Ethiopia (Demeke et al., 2015). It is recommended for the hospital to keep up with this practice.

In the present study, a total of 810 drugs were prescribed for 342 patients admitted to the emergency department of HFSUH. Of these, the most commonly prescribed drugs were analgesics (125 prescriptions, 36.5%), such as Tramadol and Diclofenac, followed by antibiotics (120, 35%). This may be due to the increased prevalence of trauma and accident as causes for presentation in this setting.

### Strengths and Limitations of the Study

Although the study design, tools, and data analysis used in this study are appropriate for assessing the variable of interest, the study does have limitations. Firstly, we included only 342 samples, which is below the sample size recommended by the WHO for the conduct of this kind of study. Secondly, the study was conducted for only three months, which could not show drug utilization variation over time. Finally, as the data were collected retrospectively, we may have missed unrecorded data.

### Conclusion

On the basis of the findings from this study, antibiotics use, injection prescribing, and the number of drugs prescribed per encounter showed considerable deviation from the standards recommended by the WHO. On the other hand, generic prescribing and prescribing from the essential drug list were not found to be problematic. Hence, it is important for the hospital to design and implement a system to promote judicious antibiotics prescribing and injection medication administration.

## Data Availability Statement

The datasets are available from the corresponding author upon reasonable request.

## Author Contributions

All authors participated starting from the conception of the research idea to interpretation of the result and manuscript authorization. All authors have read and agreed to the final manuscript.

## Conflict of Interest

The authors declare that the research was conducted in the absence of any commercial or financial relationships that could be construed as a potential conflict of interest.

## References

[B1] Ahmed Alkahtani S. (2018). Drug Utilization Patterns in the Emergency Department of Najran University Hospital, Najran. J. Pharm. Pract. Community Med. 4 (1), 12–15. 10.5530/jppcm.2018.1.4

[B2] Al Balushi K. A. Al-Shibli S. Al-Zakwani I. (2013). Drug utilization patterns in the emergency department: A retrospective study. J. Basic Clin. Pharm. 5, 1–6. 10.4103/0976-0105.128226 24808681 PMC4012701

[B3] Baba Sulemana Mohammed S. A. T. (2019). Medicines prescribing pattern in northern Ghana: does it comply with WHO recommendations for prescribing indicators? Afr. J. Pharm. Pharmacol. 13, 71–75. 10.5897/AJPP2018.4981

[B4] Bachhav S. S. Kshirsagar N. A. (2015). Systematic review of drug utilization studies & the use of the drug classification system in the WHO-SEARO Region. Indian J. Med. Res. 142, 120–129. 10.4103/0971-5916.164223 26354209 PMC4613433

[B5] Bashrahil K. A. (2010). Indicators of rational drug use and health services in Hadramout, Yemen. East Mediterr Health J. 16, 151–155. 10.26719/2010.16.2.151 20799566

[B6] Cameron A. Ewen M. Ross-Degnan D. Ball D. Laing R. (2009). Medicine prices, availability, and affordability in 36 developing and middle-income countries: a secondary analysis. Lancet (London England) 373, 240–249. 10.1016/S0140-6736(08)61762-6 19042012

[B7] Cheekavolu C. Pathapati R. M. Babasaheb Laxmansingh K. Saginela S. K. Makineedi V. P. Siddalingappa (2011). Evaluation of Drug Utilization Patterns during Initial Treatment in the Emergency Room: A Retroprospective Pharmacoepidemiological Study. ISRN Pharmacol. 2011, 261585–261585. 10.5402/2011/261585 22242208 PMC3253467

[B8] Chokshi A. Sifri Z. Cennimo D. Horng H. (2019). Global Contributors to Antibiotic Resistance. J. Global Infect. Dis. 11, 36–42. 10.4103/jgid.jgid_110_18 PMC6380099 30814834

[B9] Chowdhury A. K. A. Roy T. Faroque A. B. M. Bachar S. C. Asaduzzaman M. Nasrin N. (2011). A comprehensive situation assessment of injection practices in primary health care hospitals in Bangladesh. BMC Public Health 11, 779–779. 10.1186/1471-2458-11-779 21985397 PMC3198945

[B10] Dabaghzadeh F. Rashidian A. Torkamandi H. Alahyari S. Hanafi S. Farsaei S. (2013). Medication errors in an emergency department in a large teaching hospital in tehran. Iranian J. Pharmaceut. Res.: IJPR 12, 937–942. PMC3920714 24523775

[B11] Dalen D. (2010). Safe and Effective Medication Use in the Emergency Department. Can. J. Hosp. Pharm. 63, 57–58. 10.4212/cjhp.v63i1.876

[B12] Demeke B. Molla F. Assen A. Melkam W. Abrha S. Masresha B. (2015). Evaluation of drugs utilization pattern using WHO prescribing indicators in Ayder Referral Hospital, Northern Ethiopia. Int. J. Pharm. Sci. 6 (2), 343–347.

[B13] Denny K. J. Gartside J. G. Alcorn K. Cross J. W. Maloney S. Keijzers G. (2018). Appropriateness of antibiotic prescribing in the Emergency Department. J. Antimicrobial Chemother. 74, 515–520. 10.1093/jac/dky447 PMC6337898 30445465

[B14] Desalegn A. A. (2013). Assessment of drug use pattern using WHO prescribing indicators at Hawassa University Teaching and Referral Hospital, south Ethiopia: a cross-sectional study. BMC Health Serv. Res. 13, 170. 10.1186/1472-6963-13-170 23647871 PMC3651314

[B15] Di Simone E. Giannetta N. Auddino F. Cicotto A. Grilli D. Di Muzio M. (2018). Medication Errors in the Emergency Department: Knowledge, Attitude, Behavior, and Training Needs of Nurses. Indian J. Crit. Care Med. 22, 346–352. 10.4103/ijccm.IJCCM_63_18 29910545 PMC5971644

[B16] Dilbato D. D. Kuma Z. G. Tekle-Mariam S. (1998). A baseline survey on prescribing indicators and the underlying factors influencing prescribing in Southern Ethiopia. Ethiopia. J. Health Dev. 12 (2), 87–93.

[B17] Erah P. Olumide G. O. Okhamafe A. (2003). Prescribing practices in two health care facilities in Warri, Southern Nigeria: A comparative study. Int. J. Basic. Clin. Pharmacol. 5 (6), 2496–2499.

[B18] Fatovich D. M. (2002). Emergency medicine. BMJ (Clin. Res. ed.) 324, 958–962. 10.1136/bmj.324.7343.958 PMC1122904 11964343

[B19] Federal Democratic Republic of Ethiopia Ministry of Health (2010). Health Sector Development Programme IV: 2010/13-2014/15. HSDP IV. Federal Democratic Republic of Ethiopia Ministry of Health.

[B20] Food, Medicine and Healthcare Administration and Control Authority of Ethiopia (2012a). Manual for Good Medicine Dispensing, 2nd ed. Food, Medicine and Healthcare Administration and Control Authority of Ethiopia.

[B21] Food, Medicine and Healthcare Administration and Control Authority of Ethiopia (2012b). Manual for Medicine Good Prescribing Practice, 2nd ed. Food, Medicine and Healthcare Administration and Control Authority of Ethiopia.

[B22] Food, Medicine and Healthcare Administration and Control Authority of Ethiopia (2014a). National Essential Medicine List, fifth edition Food, Medicine and Healthcare Administration and Control Authority of Ethiopia.

[B23] Food, Medicine and Healthcare Administration and Control Authority of Ethiopia (2014b). Ethiopia Standard Treatment Guidelines for General Hospital, 3rd ed. Food, Medicine and Healthcare Administration and Control Authority of Ethiopia.

[B24] Gidebo K. D. Summoro T. S. Kanche Z. Z. Woticha E. W. (2016). Assessment of drug use patterns in terms of the WHO patient-care and facility indicators at four hospitals in Southern Ethiopia: a cross-sectional study. BMC Health Serv. Res. 16, 643–643. 10.1186/s12913-016-1882-8 27832773 PMC5103396

[B25] Hwang J.-H. Kim D.-S. Lee S.-I. Hwang J.-I. (2007). Relationship between physician characteristics and their injection use in Korea. Int. J. Qual. Health Care 19, 309–316. 10.1093/intqhc/mzm030 17720691

[B26] Isah Ao R.-D. D. Quick J. Laing R. Mabadeje AFB The development of standard values for the WHO drug use prescribing indicators.ICUM/EDM/WHO.

[B27] Kaur S. Rajagopalan S. Kaur N. Shafiq N. Bhalla A. Pandhi P. (2014). Drug utilization study in medical emergency unit of a tertiary care hospital in north India. Emergency Med. Int. 2014, 973578–973578. 10.1155/2014/973578 PMC4026969 24883208

[B28] Li Y. Xu J. Wang F. Wang B. Liu L. Hou W. (2012). Overprescribing in China, driven by financial incentives, results in very high use of antibiotics, injections, and corticosteroids. Health Aff. 31, 1075–1082. 10.1377/hlthaff.2010.0965 22566449

[B29] Ofori-Asenso R. Agyeman A. A. (2016). Irrational Use of Medicines-A Summary of Key Concepts. Pharm. (Basel Switzerland) 4, 35. 10.3390/pharmacy4040035 PMC5419375 28970408

[B30] Ofori-Asenso R. Brhlikova P. Pollock A. M. (2016). Prescribing indicators at primary health care centers within the WHO African region: a systematic analysi –2015). BMC Public Health 16, 724. 10.1186/s12889-016-3428-8 PMC4993007 27545670

[B31] Patidar R. Pichholiya M. (2016). Analysis of drugs prescribed in emergency medicine department in a tertiary care teaching hospital in southern Rajasthan.

[B32] Peth H. A., JR. (2003). Medication errors in the emergency department: a systems approach to minimizing risk. Emerg Med. Clin. North Am. 21, 141–158. 10.1016/S0733-8627(02)00085-8 12630736

[B33] Reynolds L. Mckee M. (2011). Serve the people or close the sale? Profit-driven overuse of injections and infusions in China’s market-based healthcare system. Int. J. Health Plann. 26, 449–470. 10.1002/hpm.1112 22213261

[B34] Shakirat Iyabo Bello W. A. O. Ibrahim K. Bello (2016). World Health Organization Indicators for Rational Use of Drugs in a Nigerian Secondary Hospital. 6 (2), 37–47. 10.5530/rjps.2016.2.4

[B35] Sisay M. Mengistu G. Molla B. Amare F. Gabriel T. (2017). Evaluation of rational drug use based on World Health Organization core drug use indicators in selected public hospitals of eastern Ethiopia: a cross sectional study. BMC Health Serv. Res. 17, 161. 10.1186/s12913-017-2097-3 28231833 PMC5324210

[B36] Soleymani F. Godman B. Yarimanesh P. Kebriaeezadeh A. J. J. O. P. H. S. R. (2019). Prescribing patterns of physicians working in both the direct and indirect treatment sectors in Iran; findings and implications. J. Pharm. Health Serv. Res. 10, 407–413. 10.1111/jphs.12322

[B37] Vazin A. Zamani Z. Hatam N. (2014). Frequency of medication errors in an emergency department of a large teaching hospital in southern Iran. Drug Healthc Patient Saf. 6, 179–184. 10.2147/DHPS.S75223 25525391 PMC4266248

[B38] WHO G. W. H. O. (1993). “,” in How to investigate drug use in health facilities. Selected drug use indicators- EDM Research Series No. 007. World Health Organization. [Online]. Available: http://apps.who.int/medicinedocs/en/d/Js2289e/8.6.html [Accessed].

[B39] Yarmohammadian M. H. Rezaei F. Haghshenas A. Tavakoli N. (2017). Overcrowding in emergency departments: A review of strategies to decrease future challenges. J. Res. Med. Sci. 22, 23–23. 10.4103/1735-1995.200277 28413420 PMC5377968

[B40] Yousefi N. Rashidian A. Soleymani F. Kebriaeezade A. (2017). Relationship Between the Provision of Injection Services in Ambulatory Physician Offices and Prescribing Injectable Medicines. Iranian J. Pharmaceut. Res.: IJPR 16, 399–403. PMC5423265 28496493

[B41] Zeng W. Finlayson A. E. Shankar S. De Bruyn W. Godman B. J. B. H. S. R. (2015). Prescribing efficiency of proton pump inhibitors in China: influence and future directions. BMC Health Serv. Res. 15, 11. 10.1186/s12913-014-0638-6 25609265 PMC4308879

